# Regulatory T Cells Expanded from HIV-1-Infected Individuals Maintain Phenotype, TCR Repertoire and Suppressive Capacity

**DOI:** 10.1371/journal.pone.0086920

**Published:** 2014-02-03

**Authors:** Mathieu Angin, Paul L. Klarenbeek, Melanie King, Siddhartha M. Sharma, Eshia S. Moodley, Ashley Rezai, Alicja Piechocka-Trocha, Ildiko Toth, Andrew T. Chan, Philip J. Goulder, Thumbi Ndung'u, Douglas S. Kwon, Marylyn M. Addo

**Affiliations:** 1 Ragon Institute of MGH, MIT and Harvard, Boston, Massachusetts, United States of America; 2 Department of Clinical Immunology and Rheumatology, Academic Medical Center, Amsterdam, The Netherlands; 3 HIV Pathogenesis Programme, Doris Duke Medical Research Institute and KwaZulu-Natal Research Institute for TB and HIV, University of KwaZulu-Natal, Durban, South Africa; 4 Massachusetts General Hospital, Gastrointestinal Unit, Boston, Massachusetts, United States of America; 5 Department of Paediatrics, University of Oxford, Oxford, United Kingdom; 6 Massachusetts General Hospital, Division of Infectious Diseases, Boston, Massachusetts, United States of America; 7 Department of Medicine, University Medical Center Hamburg-Eppendorf, Hamburg, Germany; University of Hawaii, United States of America

## Abstract

While modulation of regulatory T cell (Treg) function and adoptive Treg transfer are being explored as therapeutic modalities in the context of autoimmune diseases, transplantation and cancer, their role in HIV-1 pathogenesis remains less well defined. Controversy persists regarding their beneficial or detrimental effects in HIV-1 disease, which warrants further detailed exploration. Our objectives were to investigate if functional CD4^+^ Tregs can be isolated and expanded from HIV-1-infected individuals for experimental or potential future therapeutic use and to determine phenotype and suppressive capacity of expanded Tregs from HIV-1 positive blood and tissue. Tregs and conventional T cell controls were isolated from blood and gut-associated lymphoid tissue of individuals with HIV-1 infection and healthy donors using flow-based cell-sorting. The phenotype of expanded Tregs was assessed by flow-cytometry and quantitative PCR. T-cell receptor ß-chain (TCR-β) repertoire diversity was investigated by deep sequencing. Flow-based T-cell proliferation and chromium release cytotoxicity assays were used to determine Treg suppressive function. Tregs from HIV-1 positive individuals, including infants, were successfully expanded from PBMC and GALT. Expanded Tregs expressed high levels of FOXP3, CTLA4, CD39 and HELIOS and exhibited a highly demethylated TSDR (Treg-specific demethylated region), characteristic of Treg lineage. The TCRß repertoire was maintained following Treg expansion and expanded Tregs remained highly suppressive *in vitro*. Our data demonstrate that Tregs can be expanded from blood and tissue compartments of HIV-1+ donors with preservation of Treg phenotype, function and TCR repertoire. These results are highly relevant for the investigation of potential future therapeutic use, as currently investigated for other disease states and hold great promise for detailed studies on the role of Tregs in HIV-1 infection.

## Introduction

CD4^+^ regulatory T cells (Tregs) have been shown to be essential for the development and the maintenance of peripheral tolerance and immune homeostasis [1]. Indeed, Treg dysfunction is associated with allergy, autoimmunity, cancer or early graft rejection [2]. In the context of infectious diseases, Tregs have the potential to limit excessive inflammatory immune responses, thereby reducing tissue damage, but can also suppress antimicrobial immune responses and promote pathogen persistence [3].

The role of Tregs during HIV-1 infection remains controversial [4], [5], [6]. During the course of HIV-1 disease progression, microbial translocation from the gut, viral factors and co-infections such as human cytomegalovirus (hCMV) have emerged as the major causes of persistent immune activation and have been associated with mortality and non-AIDS morbidity [7]. In this context, Treg activity could have a beneficial effect through suppression of generalized chronic immune activation, but also through inhibition of activated CD4^+^ T cells and subsequent control of viral replication, as demonstrated by Moreno-Fernandez et al. [8]. In contrast, Tregs may play a detrimental role through inhibition of anti-HIV-1 immune responses [9], [10], [11], [12], thus promoting HIV-1 persistence at the host's expense. HIV-1 infection appears to directly and indirectly modulate Tregs *in vivo*, as suggested by data demonstrating that individuals with chronic HIV-1 infection have higher Treg frequencies than individuals who control HIV-1 infection and healthy control subjects [13], [14]. This observation has not been fully elucidated to date, but could be explained by preferential survival, tissue redistribution, increased proliferation, or conversion of non-regulatory T cells into Tregs in chronic HIV-1 infection [4], [15].

One of the main challenges for detailed functional analyses of Tregs in HIV-1 disease and their potential for future clinical application is the paucity of the natural Treg population in human peripheral blood, where thymus-derived Treg represent roughly 1–10% of the mature CD4^+^ T cell pool [16]. This poses an even greater challenge in progressive HIV-1 infection, where chronic viral replication and immune activation contribute to profound CD4^+^ T cell loss [17]. The functional characterization of Tregs in individuals with advanced HIV-1 disease, HIV-1-infected infants, for which only very small volume samples can be obtained, or from lymphoid or mucosal tissue sites where sample size is often limited, is therefore difficult. Based on our previous data demonstrating that *ex vivo* suppressive function of freshly isolated Tregs was preserved in HIV-1 positive individuals [13], we hypothesized that functional Tregs can be expanded *in vitro* from HIV-1-infected blood and tissue with preservation of phenotype and suppressive capacity.

We here describe the successful isolation and *in vitro* expansion of functional CD4^+^ Tregs from HIV-1-infected individuals, including HIV-1 controllers, individuals with progressive untreated HIV-1 infection, small volume specimen from HIV-1-infected infants and biopsies of gut-associated lymphoid tissue (GALT). Expanded Tregs were highly suppressive and exhibited an activated Treg phenotype with high expression of Treg markers and a demethylated TSDR, suggesting functional Treg lineage as opposed to activation-induced FOXP3 expression. We believe that our findings are of high relevance for potential future therapeutic exploration of Tregs and in addition will allow for more detailed investigations into the role and function of Tregs in HIV-1 disease.

## Methods

### Study subjects

The study was approved by the Institutional Review Board of the Massachusetts General Hospital (MGH, Boston, MA) and was conducted in accordance with the MGH human experimentation guidelines. Written informed consent was obtained for all study participants.

Blood samples were drawn from 10 HIV-1 controllers with asymptomatic HIV-1 infection who maintained a plasma viremia below 300 copies/ml (median CD4 count: 779 cells/µl, inter-quartile range (IQR): 526–1,084) in the absence of antiretroviral therapy, 13 individuals with chronic untreated HIV-1 infection (median viral load: 41,800 RNA copies/ml, IQR: 6,030–118,730 and median CD4 count: 362 cells/µl, IQR: 283–616) (Table 1) and a vertically HIV-1-infected infant (age: 511 days, viral load: 1,977,540 RNA copies/ml, CD4 count: 757). Blood samples from 5 HIV-1 uninfected individuals were studied as control specimen. Gut biopsies from 1 HIV-1-negative and 3 HIV-1-infected individuals (1 on antiretroviral therapy, 2 elite controllers) were also used in this study.

**Table 1:** Summary of clinical data of the HIV-1-infected study subjects.

Patient type	PBMC/Gut Sample	HAART Treated	Age (years)	Gender	Plasma viral load (HIV RNA copies/ml)	CD4 count, (cells/µl)
Treated	Ileum	Yes	45	Female	<50	153
Controller	Duodenum, Colon	No	61	Male	<20	690
Controller	Colon	No	60	Male	<20	998
Controller	PBMC	No	50	Female	<50	425
Controller	PBMC	No	51	Female	<50	460
Controller	PBMC	No	59	Female	<50	1283
Controller	PBMC	No	56	Female	<50	1786
Controller	PBMC	No	62	Male	<50	618
Controller	PBMC	No	53	Male	<50	734
Controller	PBMC	No	62	Male	<50	825
Controller	PBMC	No	43	Male	<50	1018
Controller	PBMC	No	44	Male	164	1018
Controller	PBMC	No	61	Male	243	548
Viremic	PBMC	No	48	Male	2,274	533
Viremic	PBMC	No	57	Female	4,090	297
Viremic	PBMC	No	48	Male	4,100	271
Viremic	PBMC	No	42	Male	7,960	362
Viremic	PBMC	No	26	Male	11,349	699
Viremic	PBMC	No	39	Male	21,500	1047
Viremic	PBMC	No	51	Male	27,000	475
Viremic	PBMC	No	40	Male	41,800	898
Viremic	PBMC	No	49	Male	44,500	2
Viremic	PBMC	No	46	Male	45,700	756
Viremic	PBMC	No	49	Male	68,460	295
Viremic	PBMC	No	29	Male	169,000	369
Viremic	PBMC	No	34	Male	204,000	312
Viremic	PBMC	No	1	Male	1,977,540	757

### Isolation of T cell subsets from peripheral blood and GALT

CD4^+^ T Cell-enriched PBMC were isolated from peripheral blood by density centrifugation using the RosetteSep enrichment kit (Ficoll-Histopaque; Sigma-Aldrich and STEMCELL Technologies) and labeled with anti-CD3-PE-Cy7 (BD Pharmingen), CD4-FITC (eBioscience), CD25-APC (eBioscience), CD127-PE (BD Pharmingen).

Cryopreserved PBMC samples were stained using the same panel as described above except for the addition of an exclusion channel to select for viable cells (Invitrogen).

Pinch biopsies from HIV-positive individuals were obtained by endoscopy and an Ileum biopsy from an HIV-negative individual was obtained from a laparoscopic small bowel resection. All gut samples were provided by the Ragon Institute tissue platform. After collection, biopsies underwent two rounds of collagenase type II (Sigma-Aldrich) digestions followed by filtration [18] and were stained with the same panel as the fresh PBMC described above.

CD3^+^CD4^+^CD25^+^CD127^low^ Treg and CD3^+^CD4^+^CD25^−^CD127^+^ Tconv controls subsets were sorted on a FACS Aria cell sorter (BD Biosciences) equipped for handling biohazardous material.

### Expansion of CD4^+^ Tregs and conventional T cells (Tconv)

Tregs and Tconvs were activated with anti-CD3/anti-CD28-coated microbeads (Invitrogen) at a 1∶1 bead-to-cell-ratio. On day 2, media volume was doubled and exogenous IL-2 was added (300 U/ml, NIH Aids Research & Reference Reagent Program) [19]. On day 5, cells were counted and fresh media added. On day 7 expanded T cells were assayed for their suppressive function and the remaining cells were cryopreserved for further analysis.

### Immunophenotyping of T cell subsets by flow-cytometry

Cryopreserved expanded Tregs and Tconvs were thawed and immunostained with anti-CD3-PE-Cy7, anti-CD4-qdot655 (Invitrogen), anti-CD25-PE-Cy5 (eBiosciences), anti-CD39-FITC (eBioscience), anti-CD45RA-horizon v450 (BD Pharmingen), anti-FOXP3-PE (clone PCH101, eBiosciences), anti-CTLA4-APC (BD Pharmingen), anti-HELIOS-FITC (Biolegend). For intracellular staining, the eBioscience FOXP3 staining buffer kit was used. Dead cells were eliminated using the LIVE/DEAD® Fixable Blue Dead Cell Stain Kit (Invitrogen). Flow-cytometry data were acquired on a LSR Fortessa (BD Biosciences).

### RNA isolation and real-time RT-PCR

RNA was isolated using the RNeasy Plus Kit (Qiagen) and retro-transcribed using the SuperScript III Reverse Transcriptase (Invitrogen). Primers for FOXP3 (forward (f): 5′-CAGCACATTCCCAGAGTTCCTC-3′ and reverse (r): 5′- GCGTGTGAACCAGTGGTAGATC-3′) and IL10 (f: 5′-GCGCTGTCATCGATTTCTTC-3′ and r: 5′-ATAGAGTCGCCACCCTGATG-3′) were designed using Primer3 [20] and chosen to span an exon-exon junction. Real-time PCR was performed in a Roche Applied Science LightCycler 480 using the SYBR Green I Master kit (Roche). RNA polymerase II (f: 5′-GCATGTTCTTTGGTTCAGCA-3′ and r: 5′-GGTCATTCCACTCCCAACAC-3′) gene expression was used to normalize the data by the Pfaffl method [21].

### Epigenetic analysis and TCR sequencing

Genomic DNA was isolated from Tregs and Tconvs using the DNeasy Blood & Tissue Kit (Qiagen). Quantification of TSDR demethylation by real-time PCR was performed by Epiontis (Berlin, Germany) as previously described [22].

The TCR diversity of *ex vivo* unexpanded and *in vitro* expanded Tregs was analyzed using a next generation sequencing protocol (NGS) [23], [24]. Briefly, RNA was isolated using the RNeasy plus kit (Qiagen) and cDNA was synthesized with SuperScript III Reverse Transcriptase and oligo-dT primers (Invitrogen) [25]. Linear amplification of the cDNA was performed on a T3000 thermocycler (Biometra). The amplified samples were analyzed by NGS on the Genome Sequencer FLX (Roche) using the titanium platform. After TCR sequencing, the Vß-, Jß variants and the CDR3 were identified.

### Assessment of Treg suppressive function using CFSE proliferation assays

Cryopreserved PBMC were labeled with CFSE (Invitrogen) and cultured in the presence or absence of fresh autologous CD4^+^ Tregs or Tconvs at day 7 of expansion with anti-CD2/anti-CD3/anti-CD28 microbeads (Miltenyi Biotec) at a 1∶1 bead-to-cell-ratio. After 4 days of co-culture, cells were stained with anti-CD3-PECy7, anti-CD4-APC (BD Pharmingen) and anti-CD8-AF700 (BD Pharmingen).

### ^51^Chromium release assay

Epstein-Barr virus (EBV) immortalized B-cell lines (BCL) were established and cytotoxicity assays were performed as previously described [26], [27]. Briefly, BCL loaded with a peptide specific for the HLAB*5701-restricted HIV-Gag-epitope KF11 (KAFSPEVIPMF) were used as target cells. Targets were incubated with KF11-specific cytotoxic T cell clones at a 1∶1 ratio (Target∶Effector) with or without expanded Tregs at a 1∶1 ratio (Treg∶Effector).

### Statistical analysis

All statistical analyses were performed using Prism 5.0a (GraphPad Software). Non-parametric tests of significance were performed throughout all analyses, using Kruskal-Wallis and Mann-Whitney testing for intergroup comparisons. P values of less than 0.05 were considered significant (*:P<0.05; **: P<0.01; ***:P<0.001; ****:P<0.0001).

## Results

### Successful expansion of Tregs isolated from HIV-1 positive and negative blood donors

**Figure 1:**
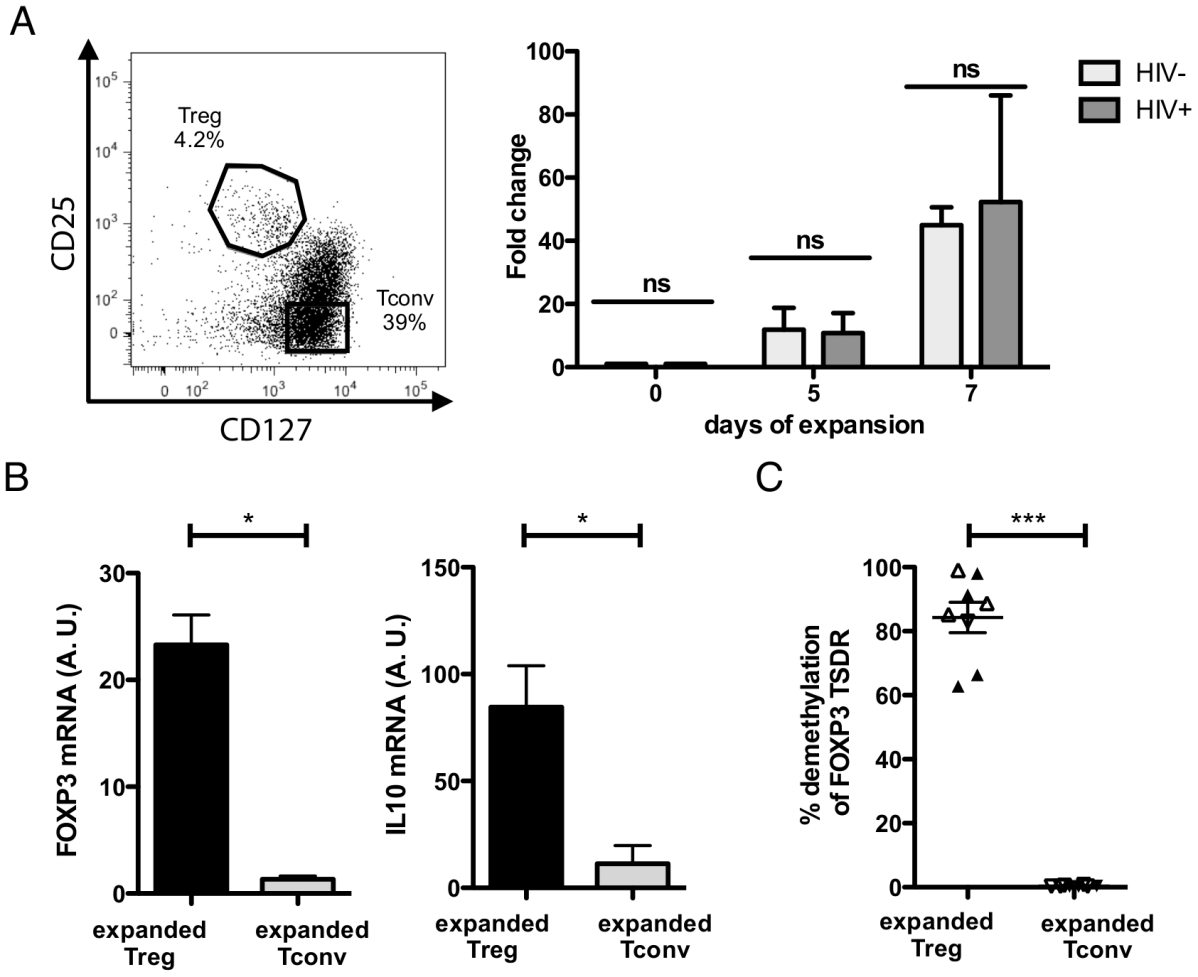
Cell sorting, FOXP3 TSDR and gene expression. A. Flow-cytometry gating strategy used to isolate CD25^+^CD127^low^ regulatory T cells (Tregs) and CD25^−^CD127^+^ conventional T cells (Tconv) from CD4^+^ T cells (Left Panel). Expansion fold change of Tregs isolated from HIV-1-infected (dark grey) (n = 8 controllers + 13 chronic untreated) and healthy (light grey) (n = 4) individuals during 7 days of cell culture (right Panel). B. Relative mRNA expression in arbitrary units (A.U.) of FOXP3 and IL-10 quantified by real time PCR in expanded Tregs (n = 3 controllers+2 chronic untreated) and Tconvs (n = 2 controllers+2 chronic untreated) isolated from HIV-1 infected individuals after 7 days of culture. C. Frequency of demethylation of the Treg Specific Demethylation (TSDR) region of the FOXP3 gene in expanded Tregs and Tconvs after 7 days of culture as assayed by real time PCR. Empty symbols represent HIV-1 controllers and solid symbols HIV-1 chronic untreated individuals.

The combination of high expression CD25 and low expression of CD127 has been described as a reliable phenotype to identify and isolate CD4^+^ Tregs [28]. Furthermore, Tregs constitutively express FOXP3, a key regulator of their development and function [29], [30], and we and others described a strong inverse correlation between CD127 and FOXP3 expression on CD4^+^CD25^hi^ T cells, including in HIV-1 positive individuals [13], [14], [28], [31]. Peripheral CD4^+^CD25^+^CD127^low^ Tregs and CD4^+^CD25^-^CD127^+^ conventional T cells (Tconvs) controls were isolated from the peripheral blood of individuals with chronic untreated HIV-1 infection, HIV-1-infected individuals with spontaneous control of HIV-1 infection (HIV controllers) and non-infected healthy donors (gating scheme Figure 1A, left). Tconvs controls underwent identical culture conditions for comparison. Isolated Tregs and Tconvs were stimulated and cultured in the presence of IL-2 for 7 days [19]. Our data show that *ex vivo* sorted Tregs from HIV-1 positive donors were successfully expanded (Figure 1A, right), with a median fold change of 49 (interquartile range (IQR): 26.4–67.7) at day 7. Treg cultures could be extended at least 19 days (data not shown), demonstrating that Tregs could be successfully expanded beyond the 7 days studied here. No significant expansion differences between individuals with spontaneously controlled, chronic untreated HIV-1 infected and healthy control subjects were observed.

### Expanded CD4^+^CD25^+^CD127^low^ T cells exhibit an activated Treg phenotype

After successful expansion of Tregs from HIV-1-infected and uninfected individuals, we next investigated and quantified the expression of selected Treg markers. Real-time PCR showed that expanded Tregs expressed high levels of FOXP3 and the suppressive cytokine IL-10 [32] compared to Tconvs expanded as controls under the same conditions (Figure 1B). In humans FOXP3 does not represent an exclusive Treg marker and can transiently be expressed by activated conventional T cells [33] to negatively regulate their proliferation and cytokine production, therefore limiting their activation state [34]. Epigenetic analysis of the *FOXP3* TSDR (Treg-specific demethylated region) using a real-time PCR assay has recently been described as a reliable method to quantify and distinguish regulatory T cells from conventional activated T cells [22]. The DNA of this region is found to be methylated in activated and resting non-regulatory T cells, while the *FOXP3* TSDR of T cells from the regulatory lineage is constitutively demethylated in this region [35]. In our study the *FOXP3* TSDR of expanded Tregs was highly demethylated, while it was found to be methylated in expanded Tconvs (Figure 1B, left). Expanded Tregs therefore revealed high expression levels of stable FOXP3, suggesting their origin derived from true functional regulatory T cell lineage.

**Figure 2:**
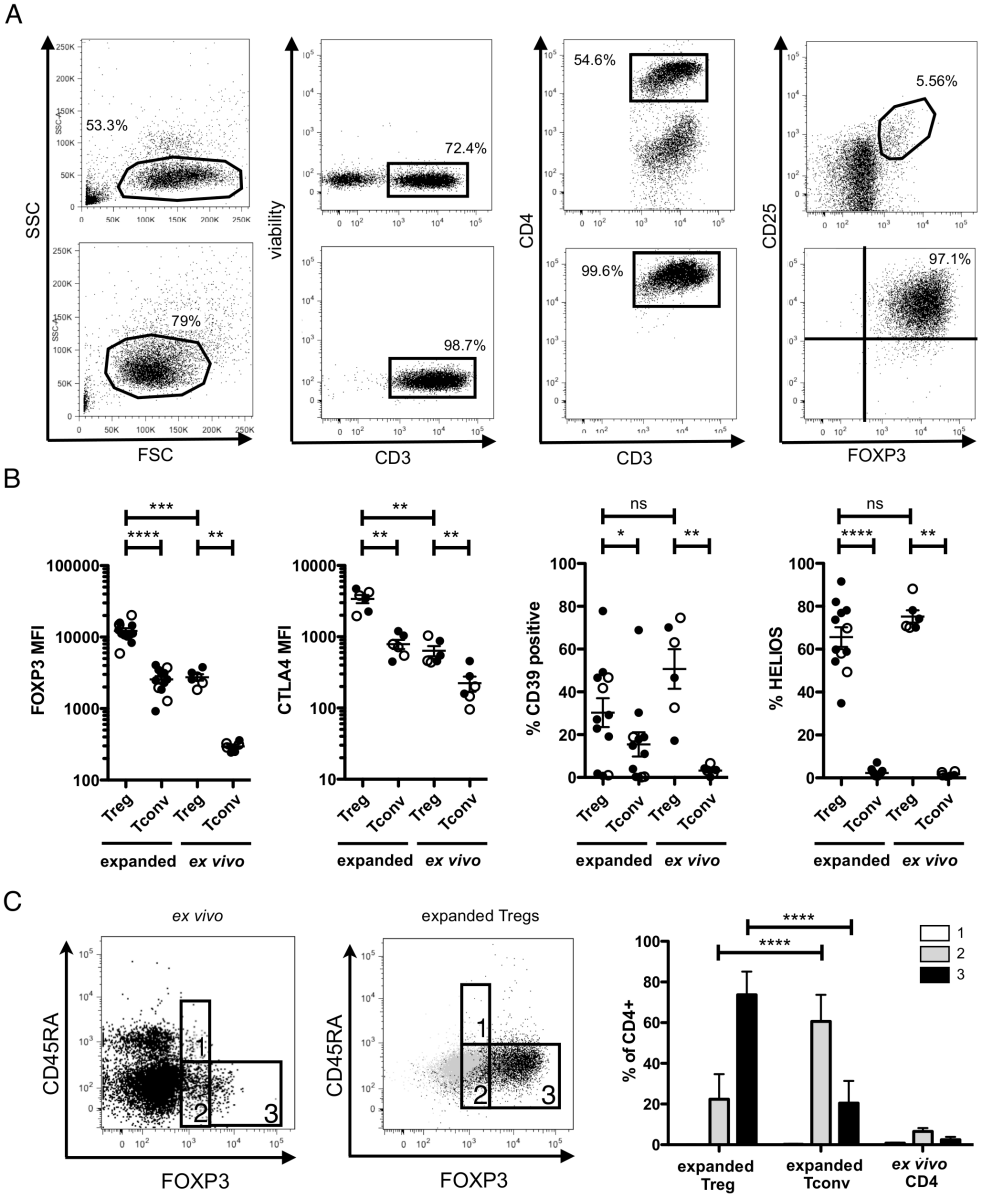
Phenotyping of expanded Tregs by flow cytometry. A. Representative examples of gating strategy used for CD25^+^FOXP3^+^ staining by flow-cytometry of *ex vivo* PBMC (upper panel) isolated from a HIV-1 controller and matched expanded Tregs (lower panel) at day 7 of expansion. B. Expression of different Tregs markers quantified by flow-cytometry of expanded (day 7) and *ex vivo* unexpanded Tregs and Tconvs. MFI = Mean Fluorescence intensity. Empty symbols represent HIV-1 controllers and solid symbols HIV-1 chronic untreated individuals. C. Representative example of flow-cytometry gating strategy used to phenotype Tregs, Tconvs (n = 3 controllers+9 chronic untreated) and *ex vivo* CD4 T cells (n = 3 controllers+3 chronic untreated) isolated from HIV-1 positive individuals based on their CD45RA and FOXP3 expression profiles [41]. The left dot plot shows *ex vivo* CD4^+^ T cells from PBMC, the middle dot plot represents an example of expanded Tregs (black dots) and Tconvs (light grey dots). The right histogram graph quantifies the different Treg subsets in HIV-1 positive individuals. Gate 1 and white columns represent “resting” CD45RA^+^FOXP3^low^ Tregs, gate 2 and grey columns represent “non-suppressive cytokine-secreting” CD45RA^−^FOXP3^low^ T cells and gate 3 and black columns represent “activated” CD45RA^−^FOXP3^high^ Tregs.

We next sought to carefully characterize the phenotype of expanded Tregs by comparing *ex vivo* unexpanded and expanded Tregs and Tconvs using flow cytometry. Examples of CD25/FOXP3 coexpression in expanded and *ex vivo* unexpanded Tregs from HIV controllers are shown in Figure 2A. While mean fluorescence intensity of CD25 expression did not differ between expanded Tregs and Tconvs (data not shown), as expected we found significantly higher FOXP3 expression in Tregs (Figure 2B). CTLA4 can transmit inhibitory signals to antigen presenting cells and is important for Treg function [36], [37]. The ectoenzyme CD39 was also shown to participate in the suppressive function of Tregs [38] and in HIV-1 infection, CD39 expression on Tregs was recently shown to correlate with disease progression [14]. Similarly to FOXP3, our data show higher CTLA4 and CD39 expression in expanded Tregs compared to conventional T cells (Figure 2B), suggesting that relative differences of CTLA4, CD39 and FOXP3 expression levels between Tconvs and Tregs were maintained after expansion. The Treg marker HELIOS has recently been suggested as a more specific marker of thymic-derived Tregs [39] and in our study, the frequency of cells expressing this molecule was high in both *ex vivo* unexpanded and *in vitro* expanded Tregs (median 72.5%, IQR: 70.1–80.6%, and median 64.8% IQR: 55.2–77.8%, respectively). HELIOS was not expressed in expanded Tconvs (Figure 2B) and the decrease of HELIOS expression found after stimulation in our culture is in line with the decreased of HELIOS-expressing FOXP3^+^ Tregs described after *in vitro* stimulation in a previous study, suggesting that obtaining large numbers of FOXP3^+^HELIOS^+^ Tregs after several rounds of stimulation may require use of a stabilizing reagent [40].

In 2009, Miyara *et al.* proposed an elegant classification scheme for the functional delineation of human CD4^+^ T cells based on the expression of FOXP3 and CD45RA [41]. Using this classification and based on *ex vivo* CD4^+^ T cell comparison as a reference, expanded Tregs showed a high amount of CD45RA^−^FOXP3^high^ activated Tregs, while expanded Tconvs were mostly constituted of CD45RA^−^FOXP3^low^ cytokine-secreting non-suppressive T cells (Figure 2C).

In summary, the high expression of FOXP3 bearing a demethylated TSDR, high CTLA4, CD39 and HELIOS as well as the CD45RA^−^FOXP3^high^ phenotype suggest that after 7 days of expansion the CD4^+^CD25^+^CD127^low^ T cells represent Tregs of an activated phenotype.

### Treg expansion did not significantly alter the TCR repertoire

**Figure 3:**
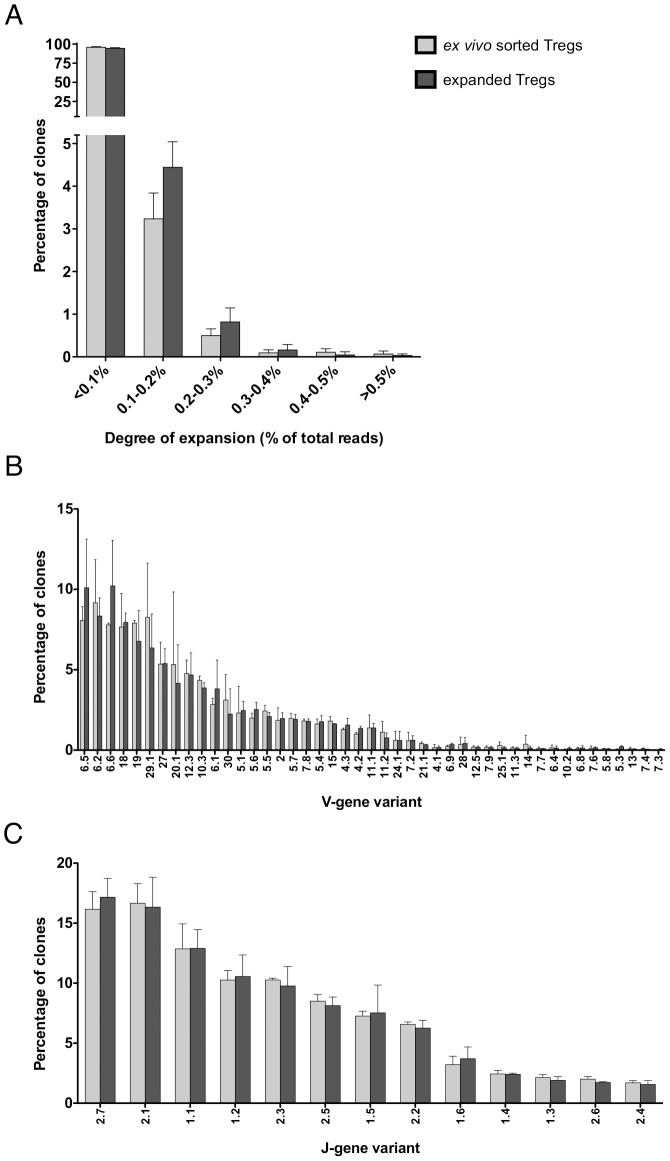
The TCR repertoire is not altered after *in vitro* expansion of Tregs. A. Degree of expansion of the TCRß repertoire (i.e. number of TCRs in a sample that belongs to an individual clone and expressed as percentage of total reads) from 2×10^4^ *ex vivo* sorted unexpanded (light grey) and 2×10^4^ *in vitro* expanded (Day 14; dark grey) Tregs isolated from the same original PBMC specimen. B. Distribution of variable-gene (Vß-gene) variants from 2×10^4^ *ex vivo* sorted unexpanded (light grey) and 2×10^4^ *in vitro* expanded (Day 14; dark grey) Treg TCR-β clones isolated from the same PBMC specimen. C. Distribution of joining-gene (Jß-gene) variants from 2×10^4^ *ex vivo* sorted unexpanded (light grey) and 2×10^4^ *in vitro* expanded (Day 14; dark grey) Treg TCR-β clones isolated from the same PBMC specimen.

After determination of the phenotype of expanded Tregs, we next investigated if *in vitro* expansion would alter T cell receptor diversity and selectively expand specific Treg clones. The next generation sequencing analysis of the Vß-CDR3-Jß region allows for identification of unique T cell clones [23]. We sequenced the Vß, Jß variants and the CDR3 regions of the TCR of *ex vivo* unexpanded and *in vitro* expanded Tregs in a subset of HIV-1-infected individuals. No specific individual clones were preferentially expanded in our study sample (Figure 3A) and the TCR-β V-(Figure 3B) and J-usage (Figure 3C) did not appear to significantly differ after expansion, suggesting that the use of anti-CD3/anti-CD28-coated beads did not significantly alter the breadth of the TCR-β repertoire. These results support work by Hoffmann et al. who found that the TCR Vβ-chain of Tregs *in vitro* stimulated with artificial antigen-presenting cells proliferated polyclonally and did not lose clonotypes [42].

### Expanded Tregs from HIV-1-infected individuals potently suppress T cell proliferation and HIV-1-specific cytotoxicity

**Figure 4:**
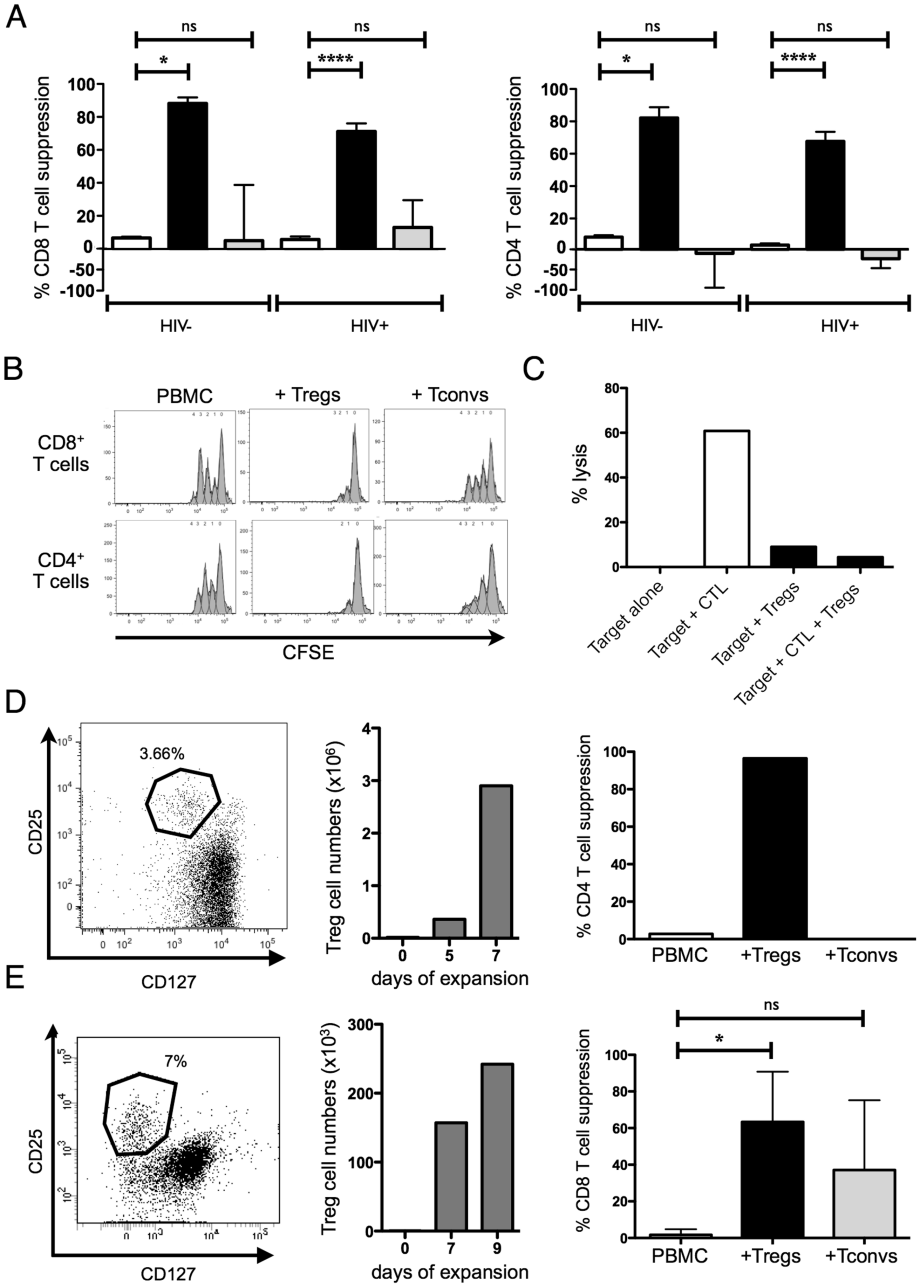
Suppressive function of expanded Tregs. A. Suppressive activity of expanded Tregs from HIV-1+ (n = 7 controllers +11 chronic untreated individuals) and healthy controls (n = 4) on activated CD8^+^ T cells (left) and CD4^+^ T cells (right). Columns represent activated T cells (white) co-cultured with autologous expanded Tregs (black) or Tconvs (grey). A suppressive activity of 100% indicates that the proliferation of activated T cells was completely inhibited and a negative suppressive activity signifies that the proliferation of T cells was higher than in the condition “T cells alone”. B. Representative example of a flow-based Treg suppressive assay after 4 days of co-culture. CFSE dilution of activated CD8^+^ T cells (upper panel) and CD4^+^ T cells (lower panel) are represented as histograms. Left columns show CFSE dilution of bead-activated T cells from frozen PBMC, the other columns represent activated T cells co-cultured with autologous expanded Tregs (middle) or Tconvs (right). C. ^51^Chromium release assay. Representation of the cytotoxic function (% lysis) of a HIV-1 specific CTL clone (effector) using a HIV-1-peptide-loaded B cell line labeled with [^51^Cr] as a target with or without expanded Tregs at a 1 Effector:1 Treg :1 Target ratio. D. Example of gating strategy used to isolate Tregs from the peripheral blood of an HIV-1-infected infant (Left). Numbers of cells counted during the expansion of these Tregs (Middle). Percentage of suppression of the expanded Tregs or expanded Tconvs on activated CD4^+^ T cells when co-cultured with autologous CFSE loaded PBMC at a 1∶1 ratio (Right). E. Example of gating strategy used to isolate Tregs from the colon of an HIV-1-infected individual (Left). The middle panel represents the numbers of cells counted during the expansion of these Tregs (Middle). Percentage of suppression of the expanded Tregs (n = 1 HIV-1-negative sample +4 HIV-1-positive samples) or expanded Tconvs (n = 1 HIV-1-negative sample +4 HIV-1-positive samples) isolated from the GALT on activated CD8^+^ T cells when co-cultured with CFSE loaded PBMC at a 1∶1 ratio (Right).

Tregs are ultimately defined through their suppressive capacity. We therefore next explored if expanded Tregs isolated from HIV-1-infected individuals remained suppressive after expansion using standardized flow-based proliferation assays [13], where CFSE-labeled activated responder cells were cultured in the presence or absence of expanded Tregs (or Tconv controls). Our data demonstrate that expanded Tregs isolated from HIV-1-positive individuals have preserved potent suppressive capacity. In contrast, no significant suppression of proliferation was observed in the presence of expanded conventional T cells (Figure 4A,B). Expanded CD4^+^ Tregs isolated from HIV-1-positive and negative individuals did not show significantly different suppressive capacities (Figure 4A,B). Expanded Tregs isolated from controllers and chronic untreated HIV-1 infected individuals were also equal in their ability to suppress T cell proliferation in this experimental system (data not shown), in line with preserved *ex vivo* Treg function in these two patient populations, as previously described [13]. Moreover when compared to our previous study [13], the suppressive function of expanded Tregs and *ex vivo* unexpanded Tregs isolated from HIV-1 positive individuals were not significantly different.

Using expanded Tregs isolated from HIV-1 positive donors, we next tested their capacity to suppress the cytolytic function of HIV-1-specific cytotoxic T lymphocyte (CTL) clones in a ^51^chromium release assay. Figure 4C shows a representative example of potent suppression by expanded Tregs of the cytotoxic activity of an HIV-1-specific CTL clone after 4 h of co-culture at a ratio of 1∶1∶1 CTL/Treg/BCL target.

We here demonstrate that Tregs expanded from HIV-1-positive individuals retain their suppressive function *in vitro*, as shown by their capacity to suppress the proliferation of activated T cells and the cytolytic activity of HIV-1-specific CTL clones.

### Expansion of Tregs from HIV-1-infected infant and gut-associated lymphoid tissue (GALT)

One of the major limitations in studying Treg biology in the context of HIV-1 infection is the limited amount of Tregs present in small volume samples. We therefore next investigated if functional Tregs can be expanded from tissue and small volume samples. Figures 4D and E show examples of Tregs isolated from the peripheral blood of an HIV-1-infected infant and from the colon of an HIV-1-infected adult. Using a flow-cytometry cell sorter we isolated 18×10^3^ and 3.5×10^3^ viable Tregs from 15×10^6^ frozen PBMC and 110×10^6^ cells isolated from fresh colonic tissue, respectively (gating is shown on Figure 4D and E, left). After 7 days, the cell number reached 2.9×10^6^ (i.e. 161 fold-change) for the Tregs isolated from the infant specimen, whereas it reached 2.4×10^5^ (i.e. 69 fold-change) after 9 days of culture of Tregs isolated from the adult GALT (Figure 4D and E, middle).

Suppressive function was quantified by flow-cytometry proliferation assays (Figure 4D and E, right) and showed that Tregs isolated from the peripheral blood of HIV-1-infected infants and the GALT of HIV-1-infected adults were functional and highly suppressive. In total Tregs from 5 gut samples (1 from a HIV-1-negative and 4 from HIV-1-positive individuals) were expanded and yielded similar results.

## Discussion

Many unanswered questions related to Tregs in the context of HIV-1 immunopathogenesis remain and it is yet incompletely understood if this cell population contributes to promotion or prevention of disease progression. Studying Tregs in CD4^+^ T cell-depleted individuals has proven to be difficult in the context of limiting cell numbers and it is unknown to date, if Tregs can be expanded from HIV-1-positive individuals for experimental or potential future therapeutic use.

In the present study we describe for the first time the successful *in vitro* expansion of CD4^+^ regulatory T cells from HIV-1 positive individuals. Expanded Tregs from HIV-1-infected donors displayed the phenotype and function of genuine regulatory T cells with a preserved TCR repertoire. Expansion of functional Tregs isolated from different blood and tissue compartments of HIV-1 patients with preserved suppressive capacity suggests that these cells are not intrinsically defective in the context of HIV-1 infection. Indeed, when comparing the expanded Tregs isolated from HIV-positive individuals (HIV controllers and individuals with chronic HIV-1 infection) and healthy control subjects, no differences in their capacity to inhibit proliferation of activated lymphocytes were observed after *in vitro* expansion. These results support our previous studies demonstrating conserved suppressive function between Tregs isolated *ex vivo* from HIV-1 positive and negative individuals [13]. However, like these previously reported *ex vivo* functional data, our results do not exclude the possibility of impairment of *in vivo* Treg function during HIV infection, e.g. in a pathologically impaired tissue microenvironment, through dysregulated interplay with antigen presenting cells such as dendritic cells [43] or loss of function as a result of direct HIV-1 infection [unpublished data], [44]. Our data also suggest that expansion of functional Tregs from HIV-infected individuals theoretically raises the possibility to use these cells therapeutically, should an appropriate clinical indication outside of their HIV disease (transplantation, autoimmune disease) arise. However, immunotherapy targeting Tregs in the context of HIV-1 infection remains controversial [45], [46] and will require further careful investigation into the role of Tregs in HIV disease.

The concept of immune silencing and potentially enhancing Treg function *in vivo* to control HIV-1-related immune activation and virus replication in conventional T cells is appealing, yet challenging to achieve. IL-2 cytokine therapy in humans promotes the generation and proliferation of effector T cells and has been shown to improve CD4 counts in HIV-1-positive individuals but not their clinical outcomes [47]. Interestingly, IL-2 treatment of HIV-1-infected patients on suppressive antiretroviral therapy resulted in the expansion of Tregs, which may have impaired the function of conventional CD4^+^ T cells [48] and could explain the overall disappointing results of this approach. Indeed, the suppressive capacity of Tregs critically depends on IL-2 [49]. In a SIV animal model, IL-2 treatment resulted again in Treg expansion but also promoted CD4^+^ T cell activation and spontaneous apoptosis [50], further highlighting the difficulties of using IL-2 *in vivo* to modulate the course of HIV-1 infection.

One alternative, but still highly experimental approach of enhancing Treg activity *in vivo* would be the transfer of autologous Tregs. In the transplantation setting, numerous animal studies described the use of polyclonally expanded autologous Tregs to induce allograft control [51], [52], [53], [54] and control autoimmune diseases [55], [56], [57]. Indeed adoptive transfer of activated Tregs provided neuroprotection in an HIV-1 encephalitis mouse model [58] and this was linked to down-regulation of proinflammatory cytokines, oxidative stress, and viral replication. However, besides technical difficulties, a major risk and challenge of isolating and expanding Tregs from HIV-1 infected donors for potential cell therapy is the re-activation of replicating virus, which needs additional careful exploration, but could potentially be managed safely in the era of HAART. Future studies should aim to reach the highest degree of Treg purity (e.g. using rapamycin alone [19] or in combination with retinoic acid [43]) and stability possible (e.g. using Oligodeoxynucleotides [39]) as adoptive transfer of activated conventional CD4^+^ T cells in the context of HIV-1 may add “fuel to the fire” in the form of new targets for the virus.

The Treg expansion approach may also be used to enrich or detect small Treg subsets such as antigen-specific Tregs [59]. Moreover, expanding functional Tregs from different tissue compartments could prove to be a useful tool to study the biology and impact of Tregs on HIV-1 infection, as the Treg TCR repertoire varies by anatomic location, presumably due to antigen encounter [60]. Tregs are important for maintenance of intestinal immune homeostasis by controlling inflammatory responses triggered by continuous antigen challenge in healthy individuals [61]. During the earliest days of HIV-1 infection, increased inflammation and immune activation occur in the gut associated lymphoid tissue [62], however little is known about the role and specificity of Tregs present in GALT and other mucosal tissues during early HIV-1 events [63], [64]. Difficult access to mucosal samples and the scarcity of the Treg population, which contribute to the lack of data, are drawbacks that could be partially overcome by Treg expansion approaches such as outlined here.

We therefore believe that this study will greatly facilitate the investigation of the role of Tregs during HIV-1 infection. A more detailed understanding of this unique T cell subset and its influence on HIV pathogenesis, immune activation and HIV-1-specific immunity will be critical for the design of potential immunotherapeutic strategies targeting Tregs (both up regulation and down regulation of Treg activity are under active investigation and consideration) [46] and possibly in the context of HIV-1 eradication [65].
